# Retinal degeneration protein 3 mutants are associated with cell-cycle arrest and apoptosis

**DOI:** 10.1038/s41420-025-02475-z

**Published:** 2025-04-15

**Authors:** Yaoyu Chen, Jens Hausmann, Benjamin Zimmermann, Simeon Oscar Arnulfo Helgers, Patrick Dömer, Johannes Woitzik, Ulrike Raap, Natalie Gray, Andreas Büttner, Karl-Wilhelm Koch, Anja U. Bräuer

**Affiliations:** 1 Division of Biochemistry, Department of Neuroscience, Carl von Ossietzky University, Oldenburg, Germany; 2 Division of Anatomy, Department of Human Medicine, Carl von Ossietzky University, Oldenburg, Germany; 3 Cancer hospital and institute of guangzhou medical university, Guangzhou, China; 4 Department of Neurosurgery, Carl von Ossietzky University, Oldenburg, Germany; 5 Research Center Neurosensory Science, Carl von Ossietzky University, Oldenburg, Germany; 6 Division of Experimental Allergy and Immunodermatology, School of Medicine and Health Sciences, Carl von Ossietzky University, Oldenburg, Germany; 7 Institute of Forensic Medicine, Rostock University Medical Center, Rostock, Germany

**Keywords:** Cancer, Oncogenesis

## Abstract

Retinal degeneration protein 3 (RD3) plays a crucial role in controlling guanylate cyclase activity in photoreceptor rod and cone cells, and mediates trafficking processes within photoreceptor cells. Loss of RD3 function correlates with severe forms of retinal dystrophy and the development of aggressive neuroblastoma cancer. In the present study, we analyzed RD3 expression in glioblastoma in comparison to non-tumor tissue using public databases and qRT-PCR. We found that RD3 is downregulated in glioblastoma compared to non-tumor tissues. To better understand the cellular function of RD3 in the context of tumor development, we performed first functional cell culture studies to clarify a possible involvement of RD3 in cell survival and the cell cycle. Interestingly, RD3 overexpression significantly decreased cell viability, which subsequently led to cell-cycle arrest at the G2/M phase and induced cell apoptosis. Conversely, single-point mutations in RD3 at the exposed protein surface involved in RD3-target interaction diminished the impact of RD3. Therefore, a controlled RD3 expression level seems to be important for a balance of cell death and cell survival rate. These new functional mechanisms of RD3 expression could help in understanding tumor development and growth

## Introduction

Retinal degeneration protein 3 (RD3) is an evolutionarily conserved protein consisting of 195 amino acids that exhibit minimal homology to other proteins [1]. In photoreceptor cells, RD3 serves as a crucial regulator of guanosine 3’, 5’-cyclic monophosphate (cGMP) synthesis via binding to retinal membrane guanylate cyclases (GC-E and GC-F), thereby inhibiting guanylate cyclase activity. In addition, the formation of a complex between GCs and RD3 facilitates the trafficking of GCs from the endoplasmic reticulum (ER) to endosomal vesicles in the retina. The GC-RD3 complex targets the light-sensitive outer segments of photoreceptors, and inhibition of cyclase activity during trafficking prevents the production of non-physiological high cGMP levels [2–6]. A nuclear magnetic resonance (NMR) spectroscopy study indicated that RD3 folds into a three-dimensional structure of a four-helix bundle showing the following arrangement: helix α1: P21–V51; α2: P75–K87; α3: P90–Q107; α4: V111–T139. Point mutations in the central helix α3 of RD3 weaken the RD3 affinity for GC-E [1, 7, 8]. Genetic deficiencies and mutations of RD3 cause early-onset photoreceptor degeneration in patients with Leber congenital amaurosis type 12 (LCA12) [4, 5, 9–11].

In addition, RD3 is expressed in other organs and tissues, including the brain, at both the transcriptional and translational level, but at a significantly lower level than in the retina [12, 13]. A more recent study compared the expression of RD3 in astrocytes and neurons with those in the retina. This study tested whether RD3 can modulate the activities of non-sensory membrane-bound guanylate cyclases GC-A and GC-B that are activated by natriuretic peptides ANP and CNP, respectively [13]. The active states of recombinant GC-A and GC-B and that of native GCs in astrocytes were inhibited by RD3 [13]. Collectively, these reports indicate that RD3 expression and function are not restricted to retinal tissue. Furthermore, one study reported that loss of RD3 correlates with the development of an aggressive neuroblastoma cancer [14]. Although these findings seem to contradict an earlier study concluding that inactivation of both *RD3* alleles in LCA12 patients does not correlate with extraocular symptoms [11], a more recent study supports the direct involvement of RD3 loss in neuroblastoma development and progression [15]. Neuroblastoma is a cancer of the nervous system that usually affects infants up to the age of six. In addition, the latter study found that intensive multimodal therapy facilitates RD3 loss in surviving cells, leading to disease progression [15]. However, any causal relationship between the abnormal regulation of RD3 expression and tumor development is unclear.

Glioblastoma (GBM) is the most aggressive and common primary brain tumor among gliomas (57.7% of total gliomas) [16, 17]. GBM usually develops within a short period of time in middle-aged people and has a poor prognosis, despite treatment with a combination of maximal surgical resection, radiotherapy, and chemotherapy [16]. Therefore, a better understanding and mapping of the molecular events involved in GBM initiation and progression is important.

In this study, we asked whether expression rates of *RD3* differ between patient cohorts suffering from GBM and healthy volunteers. To gain more insight into the regulatory features of RD3, we monitored RD3-dependent cell growth, cell-cycle arrest, and cell apoptosis using heterologous expressions of RD3 and a group of selected RD3 mutants in a common cell culture system.

## Results

### Clinical and molecular characteristics of RD3 in gliomas

**Fig. 1:**
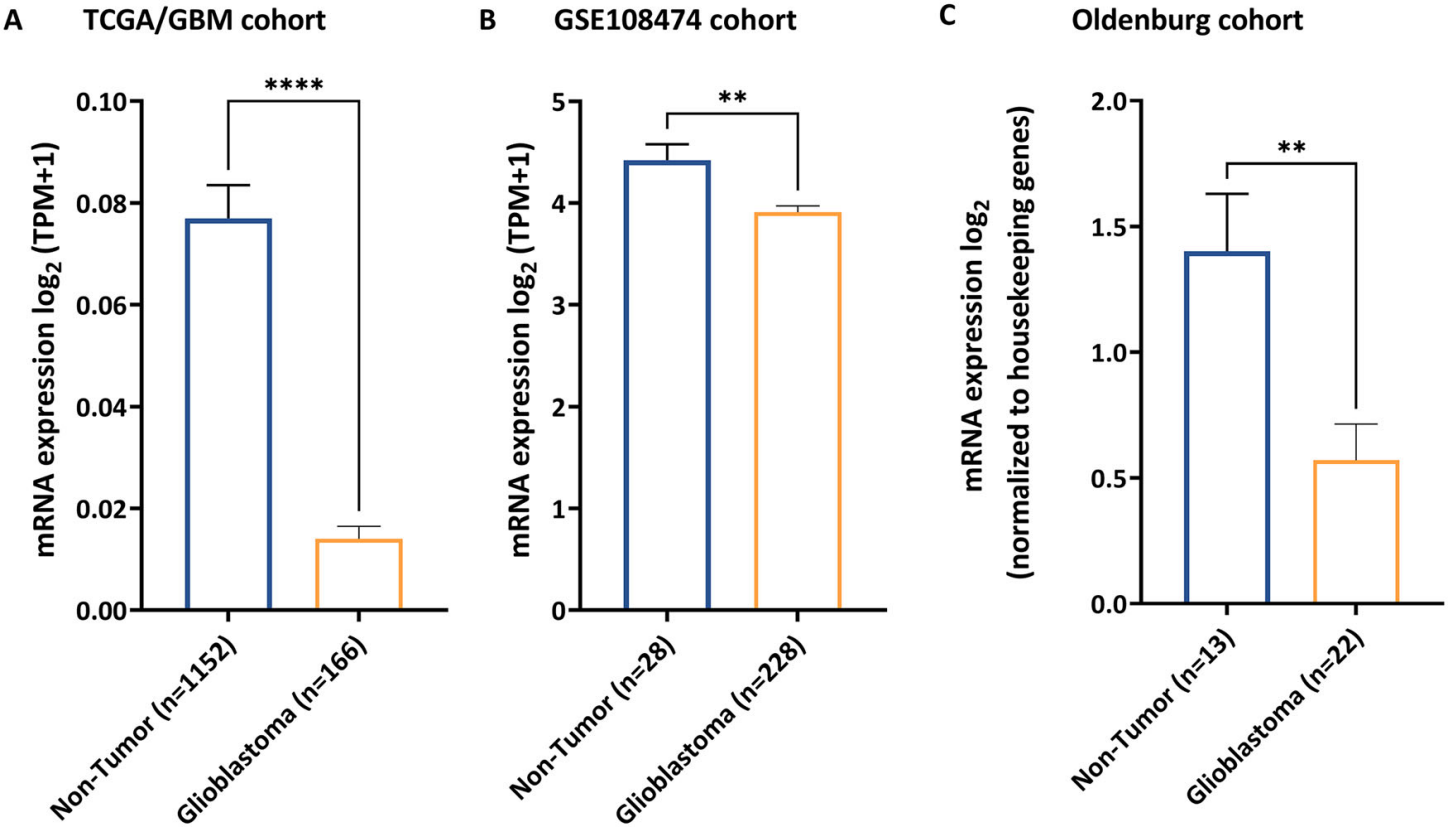
Multi-cohort differential expression analysis of RD3 in GBM. **A** The RNA-seq data were obtained from the TGCA-GBM cohort. **B** The microarray gene expression data were taken from the GSE108474 cohort. **C** GBM tissues obtained from Evangelisches Krankenhaus in Oldenburg were analyzed by qRT-PCR. All data show lower expression of *RD3* in GBM compared to non-tumor samples. The statistical analysis used an unpaired *t* test. The statistical analysis results are shown in supp. Table S5 and S6 (*P* value: * < 0.05, ** < 0.01, **** < 0.0001).

To date, there is no pathological or etiological indication that *RD3* gene expression plays a role in GBM development or disease progression, but current public datasets such as TCGA (https://www.cancer.gov/tcga) provide useful information about hypothetical correlations of *RD3* transcript levels and their impact in specific diseases. Accordingly, we first analyzed *RD3* transcript levels in the TCGA-GBM and GSE108474 cohorts. We found significant differences between non-tumor and GBM tissue (Fig. 1A, B). We then performed qRT-PCR for *RD3* expression in the GBM tissues from the Evangelisches Krankenhaus cohort and found a significant downregulation of *RD3* expression in GBM compared to non-tumor tissue (Fig. 1C, expression normalized to *TBP* and *HPRT1*).

### The clinical and prognostic significance of *RD3* expression in GBM

**Fig. 2:**
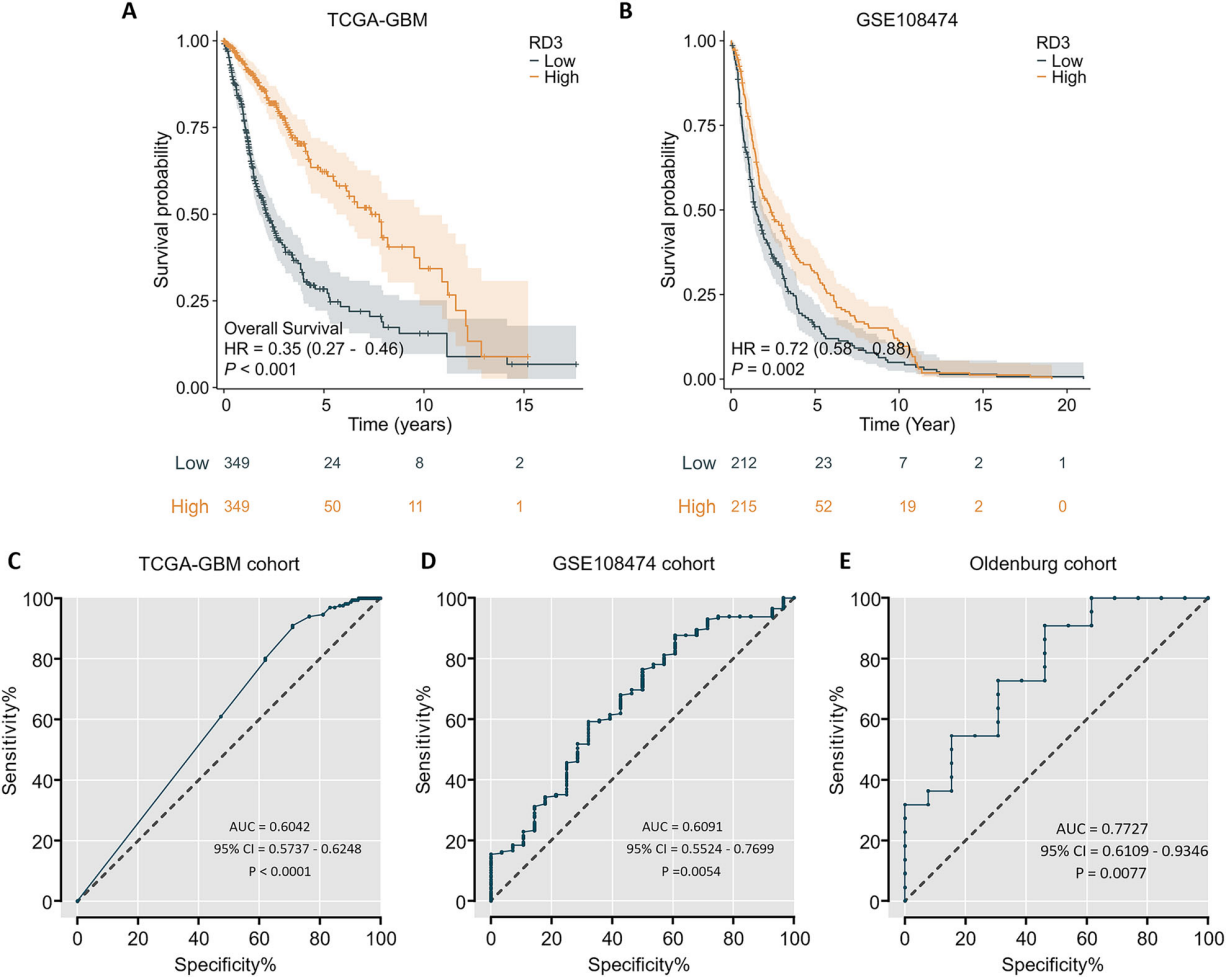
Multi-cohort Kaplan–Meier and receiver-operating characteristic (ROC) curve of RD3 in patients with GBM. **A**, **B** Overall survival-probability analysis according to *RD3* transcript level. **C**–**E** Diagnostic test of RD3 in GBM in different cohorts as indicated (AUC area under curve, 95% CI 95% confidence interval).

From our analysis, we infer that the change in *RD3* mRNA expression is associated with GBM. First, we evaluated the prognostic relevance of *RD3* transcript level in the datasets of TCGA-GBM and GSE108474. Patients were divided into *low* and *high RD3* mRNA expression subgroups according to the 50 percentiles of *RD3* expression, and overall survival curves with 95% CI (in Fig. 2A, B, the 95% confidence interval shown as a shadow), HR (hazard ratio) and *p* value were generated using Kaplan–Meier Cox regression. The results showed that in TCGA-GBM (*p* < 0.001, HR = 0.35) and GSE108474 (*p* = 0.002, HR = 0.72), patients suffering from glioma with a higher expression of *RD3* had a significantly better prognosis than the subgroup with lower expression. To evaluate the diagnostic value of *RD3* in GBM, the data from healthy donors and GBM patients of a multi-cohort study were selected for a receiver-operating-characteristic (ROC) test (Fig. 2C–E). If the area under the curve gives a parameter AUC > 0.5, the test successfully predicts whether RD3 expression correlates with a higher survival rate in GBM patients. Analysis of the datasets implemented in the current study revealed the following AUC (in brackets) for TCGA-GBM (AUC = 0.6042, Fig. 2C), GSE108474 (AUC = 0.6091, Fig. 2D), and Oldenburg cohort (AUC = 0.7727, Fig. 2E). All ROC curves had an AUC value > 0.5, indicating that the predictive ability was acceptable and significantly better than a random guess. Thus, an assessment of the predictive ability indicated that *RD3* may be a potential biomarker for the diagnosis of GBM.

### Selected point mutations in RD3

**Fig. 3:**
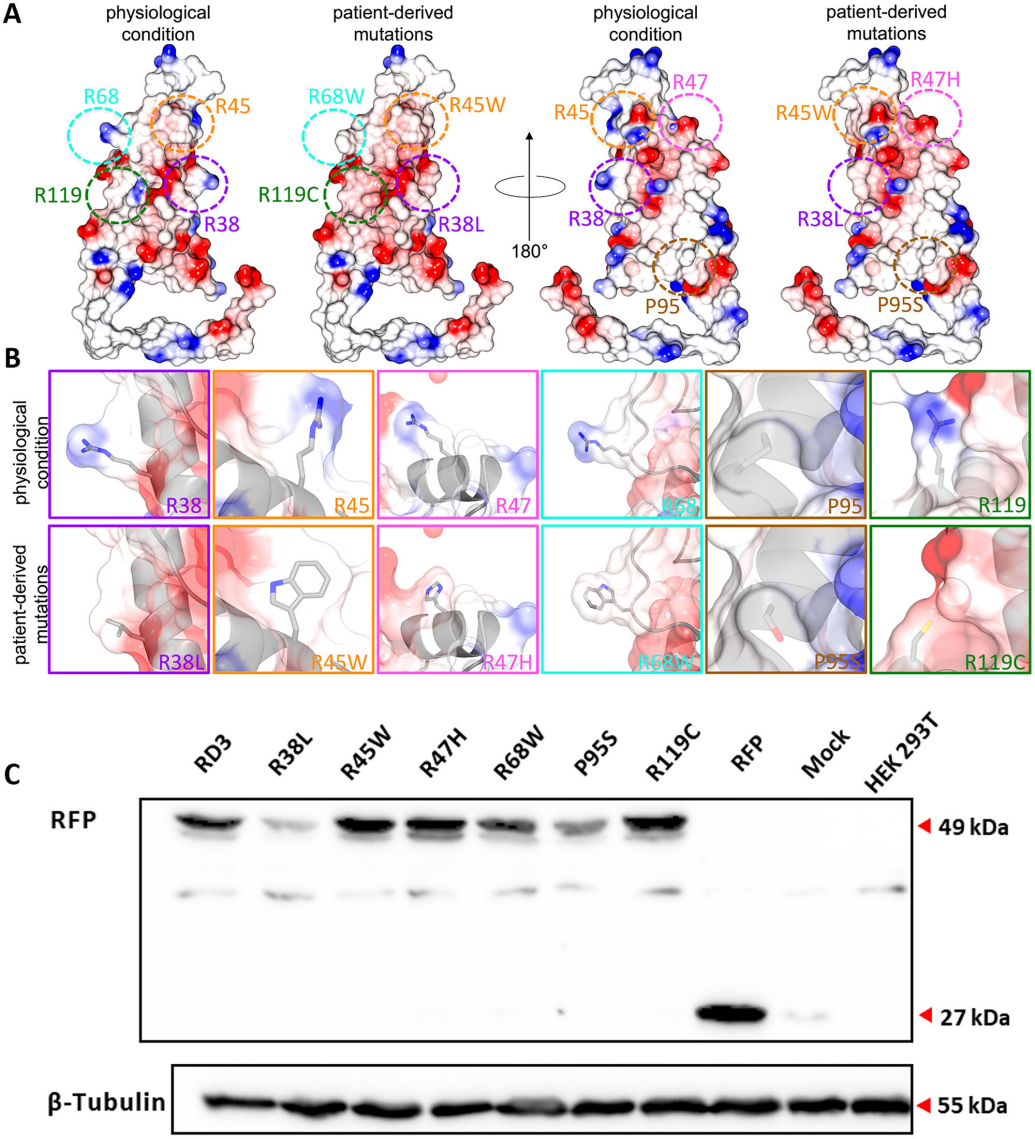
Protein structure of human RD3 based on the PDB entry 6drf. **A** Overview over RD3 structure as electrostatic surface potential representation, with residues highlighted by dashed circles in physiological and patient-derived conditions from two different perspectives. **B** Zoom onto individual residues in native (upper row) and patient-derived conditions (lower row) in RD3. The native residues and patient-derived mutations analyzed in this study are shown by their side-chain moiety and an overlay of the transparent electrostatic surface potential with secondary structure representation. **C** Western blot test of RD3 and its variants in HEK293T cell transfection. The monoclonal RFP antibody (1:2000) was used to detect the inserted RFP tag, the band around 27 kDa represents the RFP, and at 49 kDa represents the fusion protein of RD3 and RFP; the mouse β-Tubulin antibody (1:2000) was used as a housekeeping protein with a molecular mass around 55 kDa. For the original full-length blot membrane, see supp. Fig. S5.

Based on our results, we hypothesize that RD3 may have an influence on cell viability or even an indirect influence on cell cycle progression. Interestingly, we were also able to identify *RD3* gene expression in various immortalized cell lines (Fig. S1). The *RD3* positive cell lines originate not only from retinal and brain tissues, where *RD3* expression would be expected but also from human embryonic kidney tissue. In these cells, we observed the strongest expression among the analyzed cell lines. We, therefore, decided to carry out further studies with this cell line. So far, several studies describe mutations in RD3 that cause LCA12, a severe form of retinal degeneration and cell dysfunction [7, 10, 11, 17, 18]. In order to explore how *RD3* expression might interfere with cell viability in non-retinal cells, we selected amino acid positions that seem prone to functional impairment, for example, positions that are critical for the development of LCA12 (Table S1), or positions that are found in the RD3-target protein interface [19]. Based on this, we designed and obtained RD3 point mutations R38L, R45W, R47H, R68W, P95S, and R119C (Fig. 3A, B). The residue R38 in helix α1 is a recessive mutation linked to LCA12 [11]. After a structural inspection, it appears that the side-chain of R38 does not make specific contacts. However, the electrostatic surface potential is evidently swapped in R38L from a positive patch to an apolar surface region. Positions R45 and R47 were inside the solvent-exposed ends that are connected by a series of salt bridges and belong to a cluster of mutations or polymorphisms found in patients with retinal dysfunctions [4, 6]. The same is true for the residue R68. Interestingly, the guanidine group of R45 forms a salt bridge to the hydroxy group of Y60 and, additionally, a hydrogen bridge to the backbone oxygen of V58, which is inevitably impossible for the mutant R45W. Moreover, the cationic ammonium of R47 forms a salt bridge to the anionic carboxylate of E110. This interaction is absent for the variant R47H. The side-chain of R68 forms no specific interactions to other amino acid in RD3, however, this residue prominently surface-exposed (Fig. 3A, B). Residue P95 and R119 are also solvent-exposed in helix α3 and helix α4, respectively, and are present in short regions of the binding interface interacting with the target GC-E [1, 19]. The residue P95 forms three different hydrogen bridges to I97, L98, and R99, however, these are interactions that are accomplished by the backbone of RD3, and these interactions are not abolished by P95S. The side-chain of R119 makes via its guanidine group a salt bridge to the side-chain carboxylate of E177, which cannot be observed for the R119C variant.

We transfected cells with RD3 and its variants, and verified the expression of RD3 in cells by immunoblotting (Fig. 3C). Moreover, we localized RD3 and its variants by immunocytochemical assay and tested for its involvement in cellular processes. The results revealed that RD3 and its variants have a similar subcellular localization; they were detected in the cell cytosol and in vesicles. After 24 h of transfection, the nuclei of strongly red fluorescence-positive cells are in the interphase of the M phase (sup. Fig. S2). These results indicated that overexpression of RD3 and its variants might be involved, and may interfere with the cellular functions of HEK293T.

### Inhibition of cell viability by RD3 and its variants

**Fig. 4:**
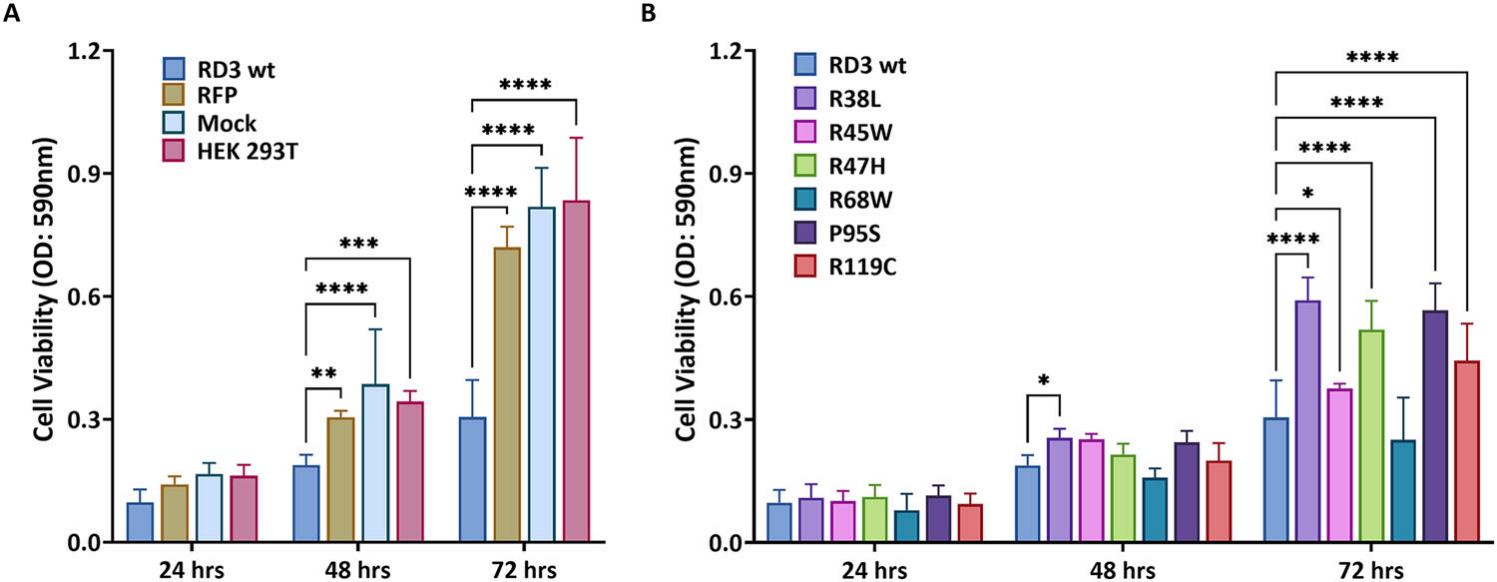
Cell-viability analysis by MTT assay at 24, 48, and 72 h after transfection of RD3 and its variants. **A** RD3 wild-type compared to control group. **B** RD3 wild type compared to variants. The statistical analysis used two-way ANOVA, and results are shown in supp. Tables S7, S8 (*P* value: *< 0.05, **< 0.01, ***< 0.001, ****< 0.0001).

The MTT assay measures cellular metabolic activity as an indicator of cell viability, proliferation, and cytotoxicity. To analyze the effects of RD3 transient overexpression on HEK293T cells, we employed this assay at 24, 48, and 72 hours post-transfection. At all examined time points, significantly reduced cell survival was observed (OD_590_ value) in cells transfected with RD3 wild-type plasmids compared to control groups (RFP empty vector, mock, and untreated cells) (Fig. 4A). After 24 hours, there were no significant differences between RD3 mutants and the RD3 wild type in subsequent measurements. However, after 48 hours, the R38L variant showed a significant improvement in cell viability. Additionally, at 72 hours, no significant differences were observed between R68W and the wild type. Conversely, variants such as R38L, R45W, R47H, P95S, and R119C exhibited more viable cells than the RD3 wild type (Fig. 4B). Compared to the control groups, all variants demonstrated a substantial negative impact on cell viability at 48 and 72 hours. For instance, while the OD_590_ value for control groups ranged ~0.7–0.8 after 72 hours, it was <0.6 for HEK293 cells transfected with RD3 variants. These findings indicated that overexpression of RD3 and its variants influences the cell viability of HEK293T.

### The mutation of RD3 is associated with cell-cycle arrest at G2/M phase

**Fig. 5:**
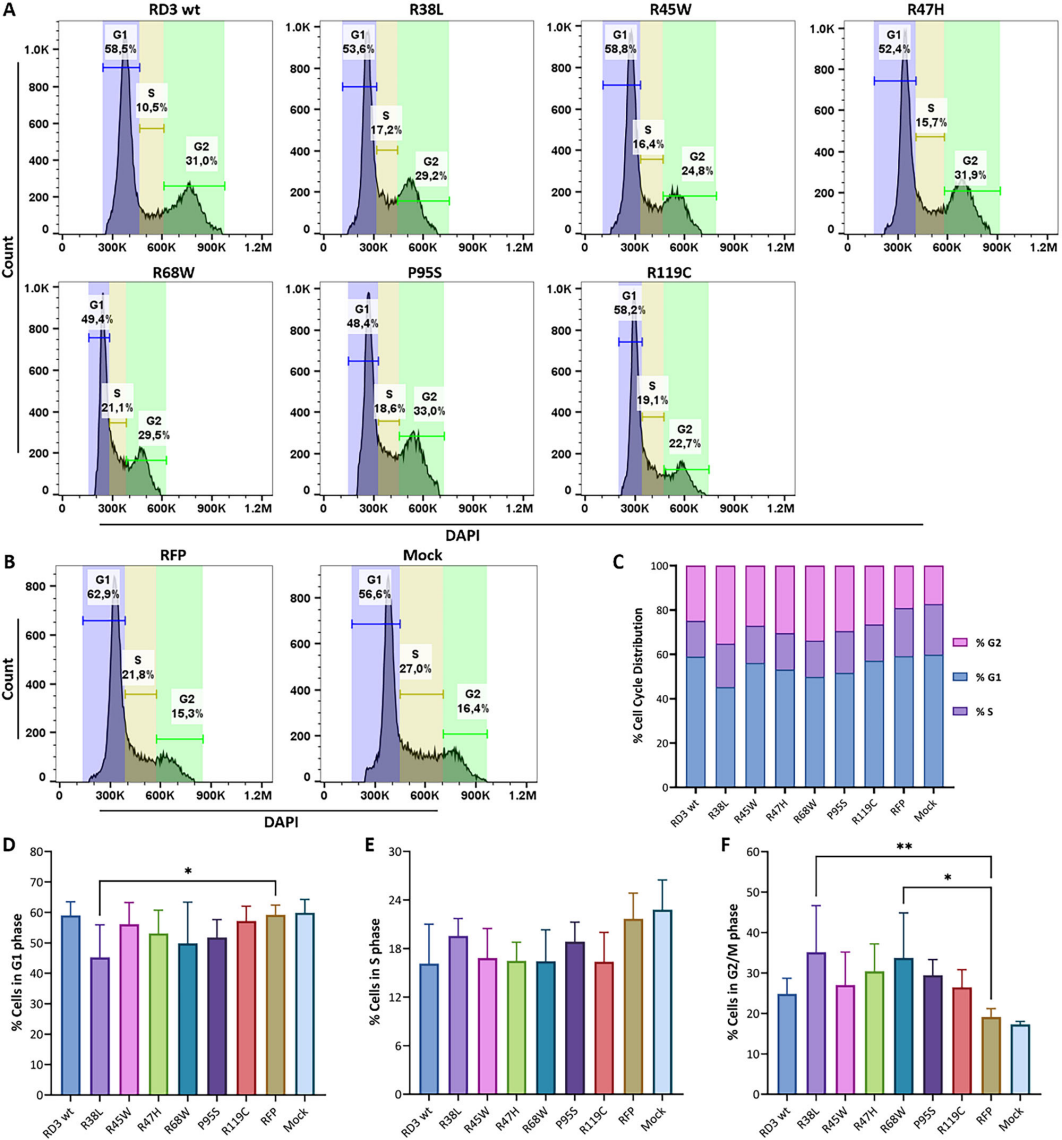
Effects of overexpression of RD3 and its variants on cell-cycle arrest in transfected HEK293T cell by using DAPI. Flow cytometry was used to detect cell-cycle distribution 24 h after transfection. **A** HEK293T cells transfected with the RD3 variants. **B** HEK293T cells transfected with empty vector and Mock control. **C**. The summary cell cycle distribution of 5 replicates. The ANOVA test was performed to analyze the percentage of cells across cell cycle **D** G1 phase, **E** S phase, and **F** G2/M phase. Statistical analysis used two-way ANOVA, and results are shown in supp. Tables S9–S11 (*P* value: *< 0.05, **< 0.01, ***< 0.001, ****< 0.0001).

Cell cycle arrest and apoptosis are crucial processes that affect cell growth; we, therefore, assessed whether transfection of HEK293T cells with RD3 and its variants regulates cell cycle progression and programmed death. To investigate how cell cycle progression would be affected by RD3 and its variant, post-transfected cells were harvested and fixed, and nuclear DNA content was measured using DAPI and fluorescence-activated cell sorting analysis. Due to the limitation of transient cell-transfection efficiency, a cell-sorting step based on the RFP tag was performed on flow cytometry (supp. Fig. S3). Compared to cells transfected with an empty RFP vector or a mock transfection, overexpression of RD3 and its variants influenced the extent of G2/M phase (Fig. 5A, B). The summary of the distribution of cell-cycle phases in five rounds of replicates showed a higher proportion of G2/M phase in the RD3 experimental group compared to the control group. In the G0/G1 phase, the R38L mutant, in particular, showed a lower proportion compared to all other experimental groups (Fig. 5C).

Subsequent statistical analysis revealed that in G1 phase cells, only R38L showed a significant decrease, while RD3 wild type, R45W, R47H, R68W, P95S, and R119C showed a slightly downregulated trend compared to controls (Fig. 5D). In S phase cells, there were no significant differences between experimental and control groups, but a moderate decrease was observed (Fig. 5E). In G2/M phase cells, R38L and R68W showed a significant increase, while the others exhibited a global upward trend compared to control RFP and mock (Fig. 5F). These results showed that two variants can disrupt the normal cell cycle of HEK293T cells by arresting these cells in the G2/M phase.

### The impact of RD3 on cell apoptosis

**Fig. 6:**
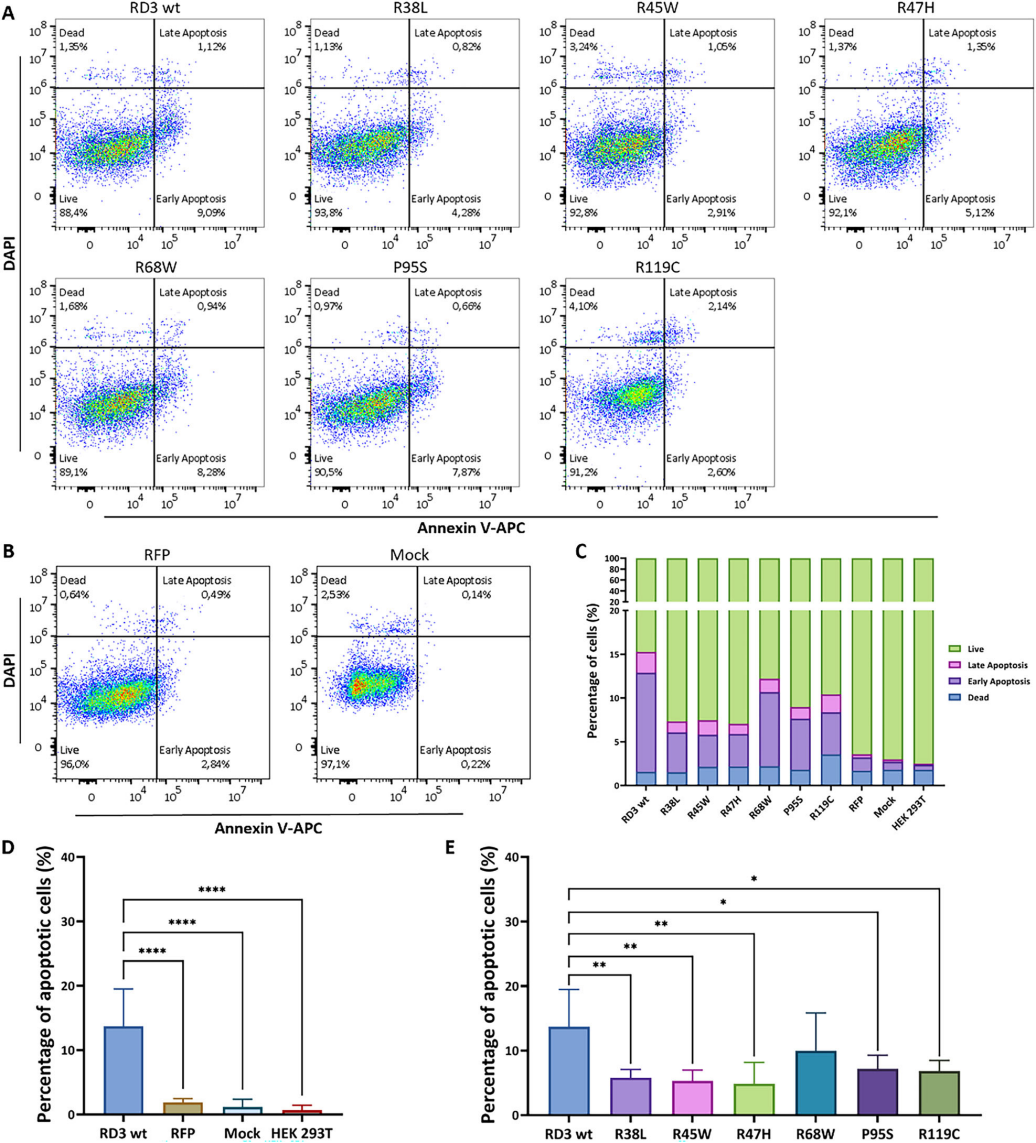
Cell programmed death analysis of RD3 and its variants transfected HEK293T cells by flow cytometry. The annexin V fuse APC and DAPI were applied for cell apoptosis detection. **A** RD3 and its variants transfected HEK293T cells. **B** Vector and Mock control. **C** Summary of cell apoptotic analysis 24 h after transfection. Apoptotic analysis of cells in **D**. RD3 and control group, **E** RD3 and its variants. The statistical analysis used two-way ANOVA, and results are shown in supp. Tables S12, S13 (*P* value: *< 0.05, **< 0.01, ***< 0.001, ****< 0.0001).

Disruption of the cell cycle by RD3 could point to an induction of apoptosis by RD3 and its point mutants. We investigated a possible induction of apoptosis in HEK293T cells, which were carefully collected after transfection and digested with 0.05% trypsin-EDTA. To detect programmed cell death, DAPI and Annexin V-APC conjugates were used for staining. Using flow cytometry, we first sorted cells by the RFP tag to find RD3 and RD3 mutant-positive cell populations (supp. Fig. S4). Positive cells were selected for measurement, while the unstained cells were used to assist the gate set separating the cell cluster of live (DAPI−, APC−), early apoptosis (DAPI−, APC+), late apoptosis (DAPI+, APC+), and death stages (DAPI+, APC−). Results in Fig. 6A, B showed the cell distribution at different stages after staining with an apoptosis indicator. It was obvious that more cells were alive in the RFP and Mock groups than in cell populations that were positive for RD3 and its variants. We applied a statistical analysis of replicate transfections yielding a summary of cell distribution, which revealed that a significant percentage of cell populations overexpressing RD3 and its variants were in the early stage of apoptosis. In comparison to control groups, these cell populations contained lower numbers of live cells and similar proportions of dead cells (Fig. 6C, D). However, the numbers of apoptotic cells overexpressing the RD3 mutants R38L, R45W, R47H, P95S, and R119C were markedly less than in cells overexpressing RD3 wild type. The mutant R68W was an exception in this regard, as it showed no significant difference to RD3 wild type (Fig. 6E).

## Discussion

The retina-specific protein RD3 controls trafficking processes in photoreceptor cells and is crucial for the controlled synthesis of the second messenger cGMP. The association of RD3 mutations in patients diagnosed with severe early-onset retinal dystrophy [10, 11, 18] brought these 195 amino acids long protein of around 22 kDa into the focus of biomedical research. The *RD3* gene was originally identified in the *RD3* mouse strain exhibiting progressive retinal degeneration [20]. Mutations in the human *RD3* gene that lead to a loss of function of the RD3 protein correlate with retinal dystrophy type LCA12 [4, 6, 7, 18].

The critical function of RD3 in health and disease apparently extends to non-retinal tissue, since recent investigations in human and mice tissues showed the expression of RD3 in different brain regions and in epithelial cells of various organs [12–15]. Loss of RD3 expression correlates with the progression of the pathogenesis in neuroblastoma and a poor survival prognosis [12, 14]. Interestingly, rare cases of vision impairment were reported as an early indicator of tumor progression in GBM patients [21–23]. Applying a data mining approach, we extended these previous studies by screening and analyzing multi-cohort datasets with respect to *RD3* expression. We thus assessed the prognostic and diagnostic value of *RD3* in glioma, particularly in GBM. Testing the predictive ability of *RD3* expression rates by an ROC test in four patient cohorts gave AUC values of at least acceptable numbers between 0.6 and 0.7 [24]. Thus, we suggest that RD3 expression could serve as a diagnostic parameter in GBM development. Our results resemble previous findings obtained from the tissue of neuroblastoma patients, in which the loss of RD3 is associated with tumor invasion, tumorsphere formation, and metastasis [14, 15]. In an animal experiment, it was shown that metastatic tumor cells that lack RD3 form a high number of aggressive metastases [14]. Furthermore, in cell culture, this group could show that re-expression of RD3 in metastatic site-derived aggressive cells results in an inhibition of their migration potential. The authors of this study suggested that RD3 functions as a metastasis suppressor, which is supported by our findings from GBM samples [14].

Cancer cells that survive intensive multimodal clinical treatment have completely lost RD3 [15], leading to a worse clinical prognosis. The mechanism of this effect remains unclear, but it demonstrates that the expression of RD3 must be set at a critical intracellular level. Furthermore, it is unclear whether the loss of RD3 is involved in causing the transformation of cells, or is an epiphenomenon triggered by processes during tumor development. Previous reports indicated that RD3 is associated with the ER stress-induced apoptosis of photoreceptor cells, but this is still poorly understood [25]. To explore a hypothetical role of RD3 in cell cycle progression, we overexpressed RD3 wild type and a set of selected RD3 mutants in HEK293 cells, and analyzed cell viability and cell growth. RD3 overexpression had a clear effect on activating the cell death program and two variants (R38L, R68W) arresting the cell cycle in the G2/M phase.

The RD3 mutants had a lower impact on cell-cycle arrest and apoptosis than RD3 wild type. We interpret this result as indicating that the effect of RD3 on cell-cycle control is specific, because locations of the point mutations are at critical positions of the interface region interacting with guanylate cyclases [10], and any disturbance might weaken or diminish the impact of RD3 on cell cycle control. Furthermore, a likely explanation for the different effects of mutants is due to alternated protein-protein interactions for the particular cellular processes (cell viability/apoptosis vs. cell cycle arrest), as our mutants are surface-exposed and, therefore, could be engaged in protein-protein interactions. A conceivable reason is that the individual mutations affect different protein-protein interfaces, which could lead to a disruption and/or enhancement of protein-protein interactions with different macromolecular interactions, and therefore, the tested cellular processes are differently influenced. In line with this, the variants R38L, R45W, and R47H show a significant increase in cell viability and consistently a significantly lower number of apoptotic cells (Figs. 4B and 6E). However, only the percentage of cells transfected with R38L significantly decreases the number of cells in G1 phase, while this mutation significantly increases the percentage of cells in G2/M phase. This effect on the cell cycle was not observed for the point mutations R45W and R47H. On the opposite, the R68W variant significantly increases the percentage of cells in G2/M (Fig. 5F) phase with no significant changes in cell viability (Fig. 4B) and apoptosis (Fig. 6E). The variants P95S and R119C significantly increase the cell viability and significantly reduce the percentage of apoptotic cells, while both mutations are not affecting the cell cycle. Based on our insights, the authors suggest that this observation could be due to alternated protein-protein interfaces. One interface could include R45, R47, P95, and R119, and a second protein-protein interface is influenced by R68. The residue R38 could be a hybrid position that bridges both interfaces, therefore cell viability, cell cycle and apoptosis are influenced. For none of the mutations, the authors can exclude that the conformational integrity of RD3 is compromised.

The interaction profile of RD3 is not restricted to photoreceptor GCs, because we showed recently that the activity of natriuretic peptide receptor GCs in astrocytes is controlled by RD3 in a fashion similar to photoreceptor cyclases [13]. This finding points to a more general role of RD3 in a signal transduction pathway involving a membrane-bound guanylate cyclase. They further indicate that the control function of RD3 may serve different cellular mechanisms in different cell types. Interestingly, there is evidence that GC-A is associated with the tumor cell cycle, apoptosis, and angiogenesis through a VEGF/GC-A/cGMP cascade [26]. VEGF has been used as a therapeutic target for the anti-angiogenesis of GBM [27], a scenario that provides a link to a signaling pathway involving the second messenger cGMP.

A direct link between the biochemical function of RD3 and cancer might also exist in its ability to regulate guanylate kinase (GUK) activity [9], an enzyme that is involved in catalyzing the 5’-GMP-to-GDP conversion [28]. GUK is an essential, ubiquitous enzyme involved in the nucleotide metabolism of cells and in diverse cellular mechanisms. Due to its crucial housekeeping role, pharmacological targeting of GUK is used in viral and cancer therapies. For example, 5’-GMP analogs serve as potent GUK inhibitors and as antiviral and anticancer prodrugs [29]. Furthermore, the loss of GUK in lung adenocarcinoma cell lines correlates with a decrease in cellular viability, proliferation, and clonogenic potential [30].

Finally, *RD3* gene is involved in the downregulation of gene expression acting in lipid metabolism, as indicated by a study of the changes in the gene expression profile of the *RD3* mouse [31]. The authors speculated that RD3 plays a central role in the metabolism of phosphatidic acid (PA), because the expression of many enzymes directly involved in lipid metabolism are dysregulated in the retina of the *RD3* mouse. Lysophosphatidic acid (LPA) is converted to PA, which is a necessary step to produce lipids important for cell membranes and chemical signaling within cells. LPA has been described as a factor in cancer progression through its stimulation of tumor proliferation and cancer-cell survival. It further increases the invasiveness of various neoplasias [32] and contributes to the development of brain malignancies [33].

In summary, we show here that low expression of the RD3 gene is a crucial factor in the development of GBM. Overexpression of RD3 impacted cell-cycle progression and triggered apoptotic pathways. Our findings extend the established roles of RD3 in photoreceptor cells to other cell types in different organs.

## Materials and methods

### Data sources and differential gene expression

The mRNA expression profiles and clinical data of GBM patients originated from The Cancer Genome Atlas (TCGA) and Gene Expression Omnibus database. The project ID, TCGA-GBM, and GSE108474 were pre-analyzed and downloaded from R2 Genomics Analysis and Visualization Platform (https://r2.amc.nl). We set up a filtering step for the cohorts taken into analysis by removing duplicated and non-clinical data. After filtering, we obtained 1318 individuals (1152 non-tumor and 166 GBM) from the TCGA-GBM cohort and 256 individuals (28 non-tumor and 226 GBM) from GSE108474. The statistically significance analysis and visualization of differential gene expression was provided by the function of one-way analysis of variance (ANOVA) and unpaired Mann–Whitney test in GraphPad Prism 7 (GraphPad Software, San Diego, CA, United States).

### Survival and ROC curve analysis

Clinical information from datasets was obtained to investigate the correlation of *RD3* mRNA expression with the prognosis of glioblastoma survival rate. Duplicated samples and those without clinical data were removed. For the overall survival analysis, we employed specific R packages (*survival, survminer, ggplot2* obtained from https://www.bioconductor.org/). The ROC tests were performed using the internal algorithms of GraphPad Prism. The area under the ROC curves (AUC) value ranging from 0 to 1 was calculated for assessing and comparing different diagnostic models.

### Patients and health donor samples

Donor samples of human brain tissue were taken from 13 individuals in the Institute of Forensic Medicine (11 males and 2 females; supp. Table S2) within 130 h after death and stored for further investigation at −80 °C. All procedures were approved by the local Ethics Committee (Rostock University Medical Center; registration ID: A2015 − 0143). None of the donors suffered from a known brain cancer disease. Human GBM specimens were freshly obtained from 22 individual surgeries of 12 males and 10 females (supp. Table S3) from the Evangelisches Krankenhaus Oldenburg (EV), with written patient informed consent (ethics registration ID: 2018-137). The tissue was snap-frozen in liquid nitrogen and stored at −80 °C. Inclusion criteria are given with the presence of a brain tumor (benign, malignant). Therapy decisions made and measures carried out or to be carried out are independent of this study, i.e., sampling. There is no exclusion criterion except for non-consent. (Age limit: 18–98 years).

### Clone construction and site-directed mutant

The pTurbo-RFP-N vector (Evrogen, Biocat, GmbH, Germany) was applied for gene expression and vector modification. The human RD3 wild-type DNA (Ensembl: ENSP00000505312.1) and its point mutants were generated using the primer listed in supp. Table S4 via polymerase chain reaction (PCR). For clone construction, Nhe I and Xho I digested the vector and PCR product. The Dephos & Ligations Kit (Merck, Darmstadt, Germany) was used for vector dephosphorylation and ligation. For constructing RD3 point mutations, the pTurbo-hRD3-RFP clone served as the template. The Q5® Site-Directed Mutagenesis Kit (New England BioLabs, Ipswich, USA) was used to generate RD3 mutants according to the manual protocol provided by the manufacturer. The plasmids with wild type RD3 and its mutants were used for transformation in E. coli cells (XL1 blue, BL21C), with antibiotic (Kanamycin) screening. The positive clones were selected for amplification and further DNA extraction. Insertions were verified by GATC Biotech (Eurofins genomics, Konstanz, Germany).

### Cell culture and transfection

HEK293T cells were cultured in Dulbecco’s modified Eagle’s medium (DMEM; Thermo Fisher Scientific) containing 10% fetal bovine serum (FBS; PAN-Biotech, Aidenbach, Germany), 2 mM L-glutamine (Merck Millipore, Darmstadt, Germany), 100 units/ml penicillin-streptomycin (PAN-Biotech) in an incubator set at 5% (v/v) CO_2_ and 37 °C. For cell transfection, cells were seeded in six-well plates at 2 × 10^5^ cells per well. The next day, METAFECTENE® liposome-based transfection reagent (Biontex Laboratories GmbH, Germany) was applied for cell transfection, in combination with the above-mentioned established plasmids according to the manufacturer’s protocol. After 24 h, the cell culture medium was changed, and the cells were processed according to the assay in use.

### Cell-viability assay

The cell viability was examined using the thiazolyl blue tetrazolium bromide (MTT) kit provided by Sigma (product number: M2128). The MTT powder was dissolved in 1 × PBS to a final concentration of 5 mg/ml and then underwent filter sterilization via a 0.22 μm filter. On the following day, HEK293 T cells were seeded into 96-well plates at a density of 1 × 10^3^ cells/well, and transfected with the relevant resources described above. Cell viability was assessed at 24, 48, and 72 h after transfection. Subsequently, 50 µl of serum-free media and 50 µl of MTT solution were added into each well, followed by 3 h incubation at 37 °C. Afterward, the 150 µl of MTT solvent solution (4 mM HCl, 0.1% NP40 in isopropanol) was used for dissolving any sediment. To accelerate the reaction, an orbital shaker was applied. Last, the absorbance at OD = 590 nm was monitored using the BioTek Epoch Microplate Spectrophotometer (Agilent Technologies, Santa Clara, USA). Four independent biological groups, each with 2 replicates, were established.

### Cell cycle detection

The transfected HEK293T cells were digested with 0.05% trypsin/EDTA at 37 °C for 5 minutes, and the DMEM medium containing FBS was used to stop the reaction. Next, the cell mixture was collected in a 15 ml Falcon tube and centrifuged at 500 × *g* for 5 min. at 4 °C. Afterwards, cells were washed twice with 5 ml of cold 1× PBS, the supernatant was discarded, and the cells were resuspended with 500 µl of fresh, cold PBS. Cell suspensions were then transferred to a new 15 ml Falcon containing 4.5 ml of ice-cold 70% ethanol. The mixture was incubated at 4 °C overnight, then centrifuged at 1000 × *g* for 5 minutes and washed with 5 ml of PBS. Cell pellets were resuspended for 10 min. in 300 µl of DAPI/TritonX-100 solution, containing 10 µl of 1 mg/ml DAPI and 0.1% (v/v) TritonX-100 in 10 ml of PBS. Five independent cell-cycle replicates were analyzed using the Cytoflex S flow cytometer (Beckman Coulter, CA, USA) on the cells transfected with red fluorescence protein. The results were analyzed with FlowJo v10.8.1 (BD Life Sciences, Ashland, USA), and the gate setting is shown in supp. Fig. S3.

### Cell apoptosis measurement

The transfected HEK293T cells were detached from the six-well plates using 0.05% Trypsin-EDTA and then transferred to a 15 ml falcon tube. Following this, the cell mixture was centrifuged at 500 × *g* for 5 min. and the supernatants were discarded. The cells were washed with cold 1× PBS, and after the washing step, the cell pellet was resuspended in 300 μl binding buffer (10 mM HEPES, 150 mM NaCl, 2.5 mM CaCl_2_•2H_2_O). Cells were counted by aspirating ~2.5 × 10^5^ cells for Annexin V-APC Conjugates,0.1 µg/ml concentration, (Thermo Fisher Scientific, Waltham, MA, USA) and DAPI staining, with a final volume of 50 µl, as recommended by the manufacturer. Cells were incubated at room temperature for 15 mins, 300 µl of binding buffer was added, briefly vortexed, and loaded onto the Cytoflex S (Beckman Coulter, CA, USA) flow cytometer for measurement. The results were analyzed using FlowJo v10.8.1[30-day free trial] (BD Life Sciences, Ashland, USA); the gate setting is shown in supp. Fig. S4.

### qRT-PCR

The RNA of human brain tissues from 13 healthy donors (supp. Table S2), and 22 GBM patients (supp. Table S3) were homogenized in TRIzol^TM^ reagent (Thermo Fisher Scientific, Waltham, MA, USA) and extracted according to the manufacturer’s protocol. The same protocol was also used for RNA isolation from the cell lines. The following cell lines were examined for the expression of *RD3*: HEK293T/ HEK293H, human embryonic kidney cells (RRID: CVCL:0063/ RRID: CVCL_6643); 661 W, mouse retinal cone cells (RRID: CVCL_6240); B6-RPE07, mouse retinal pigment epithelial cell (RRID: CVCL_DF62); Neuro-2a, mouse neuroblast derived from the neural crest (RRID: CVCL_0470); HT-22, mouse hippocampal cells (RRID: CVCL_0321).

After RNA concentration measurement using BioSpectrometer basic (Eppendorf, Hamburg, Germany), the 0.5 μg RNA was applied for cDNA synthesis by using a high-capacity cDNA reverse-transcription kit from Thermo Fisher Scientific. The transcript level of RD3 measurements was based on TaqMan^TM^ Fast Universal PCR Master Mix, No AmpErase^TM^ UNG (Thermo Fisher Scientific, Waltham, MA, USA) on hard-shell 96-Well PCR plates from Bio-Rad Laboratories (Hercules, CA, USA), and TaqMan probes. The human *RD3* probe, and two housekeeping *TBP* and *HPRT1* probes were purchased from Thermo Fisher Scientific (RD3: Hs01650935_m1, TBP: Hs00427620_m1, and HPRT1: Hs01003270_g1). For mouse cell lines mouse *RD3* probe and one housekeeping gene *ß-actin* were also purchased from Thermo Fisher Scientific (RD3: Mm00660322_m1, mouse *ß-actin* Mm00607939_s1).

### Structural analysis

We used the PDB entry 6drf as a model for our structure analysis [1]. Structural mutants were generated virtually with Coot and also analyzed with the aforementioned program [34]. Structural figures have been prepared with CCP4mg [35].

### Western blot

Protein fractions from HEK293T cells were incubated with 5× Laemmli buffer containing 1% (v/v) β-mercaptoethanol at 95 °C for 5 min and analyzed by SDS-PAGE with 12% acrylamide. Immunoblotting was performed using a 0.45 μm nitrocellulose (NC) membrane and semi-dry blotting system. After blotting at 200 mA for 30 min, the membrane was blocked in 5% milk powder (Carl Roth) in TBST at room temperature for 1 h. Primary anti-mouse-RFP antibody (MA5-15257, Thermo Fisher Scientific) was incubated overnight at 4 °C in blocking solution at a dilution of 1:2000. The next day, the membrane was washed with TBST, then incubated with horseradish peroxidase-conjugated secondary antibodies (GE Healthcare, Boston, MA, United States) at a dilution of 1:10,000 in blocking solution. The blot was washed again, and the immunoreaction was detected using Clarity or Clarity Max ECL substrate (Bio-Rad Laboratories, Hercules, CA, United States) according to the manufacturer’s protocol.

### Immunocytochemistry

The HEK293T cells were washed using 1× PBS and, following 24 h of transfection with pTurbo-hRD3-RFP plasmid, fixed with ice-cold 4% paraformaldehyde (Merck KGaA, Darmstadt, Germany) and 15% sucrose in 1× PBS for 20 minutes. Afterward, the fixed cells were washed three times with 1× PBS for 10 min and permeabilized in 0.1% TritonX-100/PBS + 0.1% sodium citrate for 3 min. Cells were washed three times again with 1× PBS and blocked in a buffer containing 10% FCS/1% NGS/PBS for 1 h. DAPI was applied for staining the nuclei, and coverslips were again washed three times for 10 min with 1× PBS, and cells were mounted with Immu Mount Vectashield Hard Set Mounting medium (Vector Laboratories, Burlingame, CA, USA). Imaging of the mounted slides was performed using an Olympus FV3000 confocal microscope.

### Statistical analysis

RNA-seq and microarray data from TCGA-GBM and GSE108474 were analyzed using one-way ANOVA (Fig. 1A, B), while data from qRT-PCR were analyzed using unpaired Welch’s *t* test (Fig. 1C). The ROC curve (Fig. 2A–E) was processed using the algorithm provided by GraphPad Prism 7 (GraphPad Software, San Diego, CA, United States). For multiple comparisons (Figs. 4, 5D–F and 6D, E), the two-way ANOVA of GraphPad Prism was used.

## Supplementary information

supp. Fig S1-S5

supp. Table S1-S13

## Data Availability

The datasets for this study are stored on a data repository of the University of Oldenburg can be obtained on request.
