# Supplementary material for: Retinal degeneration protein 3 mutants are associated with cell-cycle arrest and apoptosis
Source: Cell Death Discov. 2025 Apr 15;11:175. doi: 10.1038/s41420-025-02475-z (PMC12000573; doi:10.1038/s41420-025-02475-z)
Supplement: Supplementary file 1 — supp. Fig S1-S5 [file 41420_2025_2475_MOESM1_ESM.docx]

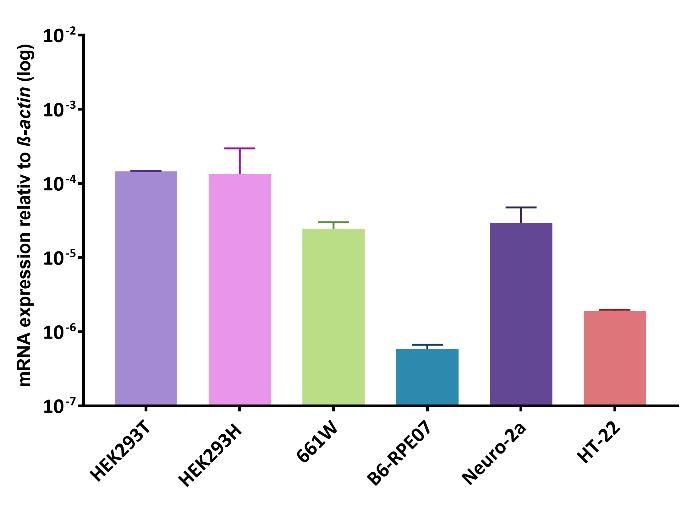


**Supplemental Figure S1:** Quantitative RT-PCR analyses of RD3 expression from HEK293T/ HEK293H, human embryonic kidney cells, 661W, mouse retinal cone cells, B6-RPE07, mouse retinal pigment epithelial cell, Neuro-2a, mouse neuroblast derived from the neural crest, HT-22, mouse hippocampal cells revealed highest expression in HEK293 cell lines compared to the others, all normalized to *ß-actin* expression. Notably, the highest expression in cell lines corresponds to the low expression of RD3 in the mouse brain. The mouse retina shows a 1000-fold higher expression [13].
