# Supplementary material for: Retinal degeneration protein 3 mutants are associated with cell-cycle arrest and apoptosis
Source: Cell Death Discov. 2025 Apr 15;11:175. doi: 10.1038/s41420-025-02475-z (PMC12000573; doi:10.1038/s41420-025-02475-z)
Supplement: Supplementary file 2 — supp. Table S1-S13 [file 41420_2025_2475_MOESM2_ESM.docx]

**Supplemental Table S1: Summarizing information of RD3 variants and their potential links to diseases**

| **Amino acid change** | **Potential link to disease form** | **References and links to databases** |
| --- | --- | --- |
| **p.R38***  premature stop codon | Leber congenital amaurosis 12;  Ovarian Epithelial Tumor | Perrault et al. (2013) https://www.ncbi.nlm.nih.gov/clinvar/variation/189792/ https://depmap.org/portal/gene/RD3?tab=characterization&characteriz ation=mutation |
| **p.R38L** | Papillary renal cell carcinoma; | https://cancer.sanger.ac.uk/cosmic/mutation/overview?id=112398541 https://bit.ly/3QK3tn2 |
| **p.R45W** | Endometrioid carcinoma;  Adenocarcinoma | https://cancer.sanger.ac.uk/cosmic/mutation/overview?id=112398773  https://bit.ly/3QK3tn2 |
| **p.R47H** | Leber Congenital Amaurosis 12 (variant of uncertain significance)  Adenocarcinoma | https://www.ncbi.nlm.nih.gov/clinvar/variation/935765/  https://cancer.sanger.ac.uk/cosmic/mutation/overview?id=112398074  https://depmap.org/portal/gene/RD3?tab=characterization&characteriz  ation=mutation  https://bit.ly/3QK3tn2 |
| **p.R68W** | Leber Congenital Amaurosis 12  Endometrioid carcinoma  Adenocarcinoma | Friedman et al. (2006)  https://www.ncbi.nlm.nih.gov/clinvar/variation/466320/ https://cancer.sanger.ac.uk/cosmic/mutation/overview?id=112397909 |
| **p.P95S** | Leber Congenital Amaurosis 12 (variant of uncertain significance)  Medulloblastoma  Small Cell Lung Cancer | https://www.ncbi.nlm.nih.gov/clinvar/variation/838670/ https://depmap.org/portal/gene/RD3?tab=characterization&characteriz ation=mutation  https://bit.ly/3QK3tn2 |
| **p.R119C** | Leber Congenital Amaurosis 12 (variant of uncertain significance)  Adenocarcinoma | https://www.ncbi.nlm.nih.gov/clinvar/variation/1045675/ https://cancer.sanger.ac.uk/cosmic/mutation/overview?id=112398638 |
